# ﻿A new species and four new records of *Bacidia* (Lecanorales, Ramalinaceae) from South Korea, with a key to Korean species

**DOI:** 10.3897/mycokeys.93.89283

**Published:** 2022-10-19

**Authors:** Beeyoung Gun Lee, Jae-Seoun Hur

**Affiliations:** 1 Baekdudaegan National Arboretum, Bonghwa, 36209, Republic of Korea Baekdudaegan National Arboretum Bonghwa Republic of Korea; 2 Korean Lichen Research Institute, Sunchon National University, Suncheon 57922, Republic of Korea Sunchon National University Suncheon Republic of Korea

**Keywords:** biodiversity, corticolous, lichen, phylogeny, taxonomy

## Abstract

A new species, *Bacidiafuscopallida* Lee & Heo and four new records, *B.ekmaniana* R. C. Harris, Ladd & Lendemer, *B.friesiana* (Hepp) Körb., *B.heterochroa* (Müll. Arg.) Zahlbr. and *B.suffusa* (Fr.) A. Schneid., are described from South Korea. *Bacidiafuscopallida* differs from *B.diffracta* S. Ekman, the most similar species, by warted but non-granular thallus, paler and smaller apothecia without pruina, proper exciple without crystals, over 11-septate ascospores and smaller pycnidia and pycnoconidia. *Bacidiaekmaniana* is recorded new to Asia, *B.heterochroa* is reported new to northeastern Asia and *B.friesiana* and *B.suffusa* are new to Korea. Molecular analyses employing internal transcribed spacer (ITS) sequences strongly support the classification of the five species of *Bacidia*. A surrogate key is provided to assist in the identification of all 19 taxa in *Bacidia* of Korea.

## ﻿Introduction

*Bacidia* has become a species-rich genus since De Notaris’ (1846) introduction. *Bacidia* (230 spp. including *Bacidiopsora*) is one of the largest genera in Ramalinaceae, with *Ramalina* (230 spp.) (Wijayawardene et al. 2020). The genus *Bacidia* was defined in a wide sense by the characteristics of crustose lichens with a chlorococcoid photobiont, biatorine or lecideine apothecia, 8-spored asci with colourless and transversely 3- or more septate ascospores (Zahlbruckner 1905, 1921–1940). However, the traditional characterisation of the genus has been considered coarse and unnatural. The genus has been split (e.g. Santesson 1952; Vězda 1978) and particularly new taxonomic applications, based on ascus structures (Hafellner 1984), excipulum structures (Vězda 1990) and molecular results (Ekman and Wedin 2000; Ekman 2001) have reclassified the large genus into tens of different genera (e.g. Vězda 1986; Sérusiaux 1986, 1993, 1995; Lücking 1992, 1995; Aptroot and Sipman 1993; Lücking et al. 1994; Ekman 1996; Kistenich et al. 2018). Ekman (2001) represented that *Bacidia* might be delimited to the *B.rosella* (Pers.) De Not., the type species, group in a strict sense (Brand et al. 2009) and most *Bacidia* species with blue-green pigment in epihymenium are closer to *Toninia* than the type species group, based on molecular phylogeny although *B.schweinitzii* (Fr. ex E. Michener) A. Schneid.) can be an exception.

*Bacidia* is one of the least explored genera in Korea and the genus has just been reported since the 2010s. Since Joshi et al. (2011) introduced *B.arceutina* (Ach.) Th. Fr., *B.schweinitzii* and *B.subincompta* (Nyl.) Arnold (syn. *Toniniopsissubincompta* (Nyl.) Kistenich, Timdal, Bendiksby & S. Ekman), overall 18 species have been recorded in Korea (Zhang et al. 2012; Aptroot and Moon 2014, 2015; Kondratyuk et al. 2016, 2017, 2019a, b; Liu 2018; Yakovchenko et al. 2018). Although detected on diverse substrates (e.g. bark, moss, rock or artificial wood fence), they are mainly corticolous and were collected on deciduous, wide-leaved tree barks in humid forests.

**Figure 1.**
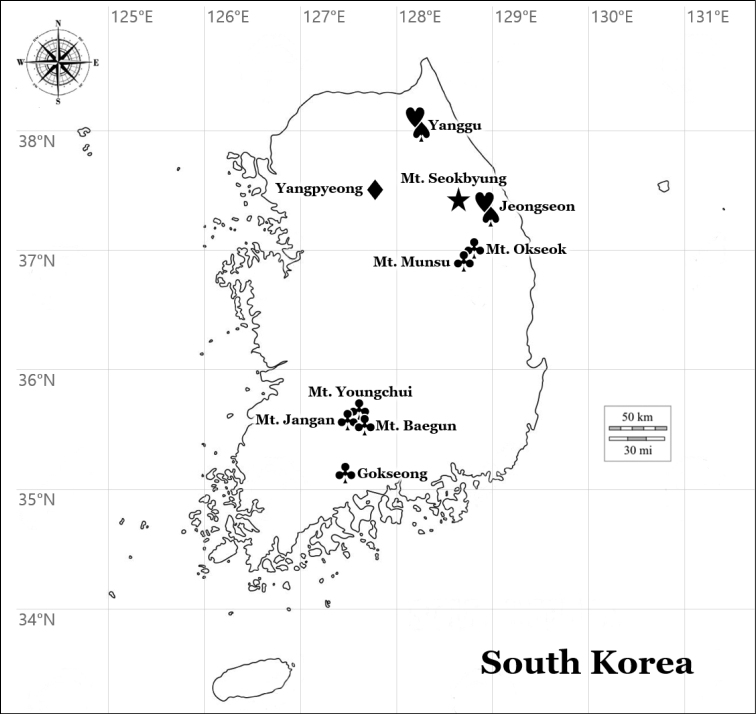
Specific collection sites (black symbols) for the new species *Bacidiafuscopallida* (black star) and four new records, *B.ekmaniana* (black club), *B.friesiana* (black diamond), *B.heterochroa* (black hearth) and *B.suffusa* (black spade).

This study describes a new species and four new records of the lichen genus *Bacidia*. Field surveys for the lichen biodiversity in the main mountains of Korea, i.e. Baekdudaegan, and several forested wetlands of South Korea were carried out during the spring to summer of 2019–2021 and 54 specimens of *Bacidia* were collected from barks of deciduous wide-leaved trees and shrubs (Fig. 1). The specimens were comprehensively analysed and identified as a new species, *B.fuscopallida*, and four new records, *B.ekmaniana*, *B.friesiana*, *B.heterochroa* and *B.suffusa*. All the collected specimens are deposited in the Herbarium of the Baekdudaegan National Arboretum (KBA), South Korea.

## ﻿Materials and methods

### ﻿Morphological and chemical analyses

Hand sections were prepared manually with a razor blade under a stereomicroscope (Olympus optical SZ51; Olympus, Tokyo, Japan), examined under a compound microscope (Nikon Eclipse E400; Nikon, Tokyo, Japan) and pictured using a software programme (NIS-Elements D; Nikon, Tokyo, Japan) and a DS-Fi3 camera (Nikon, Tokyo, Japan) mounted on a Nikon Eclipse Ni-U microscope (Nikon, Tokyo, Japan). The ascospores were examined at 1000× magnification in water. The length and width of the ascospores were measured and the range of spore sizes was shown with average, standard deviation (SD), length-to-width ratio and the number of measured spores. Thin-layer chromatography (TLC) was performed using solvent system C according to standard methods (Orange et al. 2001).

### ﻿Isolation, DNA extraction, amplification and sequencing

Hand-cut sections of ten to twenty ascomata per collected specimen were prepared for DNA isolation (Table 1) and DNA was extracted with a NucleoSpin Plant II Kit in line with the manufacturer’s instructions (Macherey-Nagel, Düren, Germany). PCR amplifications for the internal transcribed spacer region (ITS1-5.8S-ITS2 rDNA) RNA genes were achieved using Bioneer’s AccuPower PCR Premix (Bioneer, Daejeon, Korea) in 20-μl tubes with 16 μl of distilled water, 2 μl of DNA extracts and 2 μl of the primers ITS5 and ITS4 (White et al. 1990). The PCR thermal cycling parameters used were 95 °C (15 sec), followed by 35 cycles of 95 °C (45 sec), 54 °C (45 sec) and 72 °C (1 min) and a final extension at 72 °C (7 min), based on Ekman (2001). The annealing temperature was occasionally altered by ±1 degree in order to obtain a better result. PCR purification and DNA sequencing were accomplished by the genomic research company Macrogen (Seoul, Korea).

**Table 1.** Hand-cut section information for DNA isolation.

Species	*Bacidiafuscopallida*	*Bacidiaekmaniana*	*Bacidiafriesiana*	*Bacidiaheterochroa*	*Bacidiasuffusa*
Specimens	KBA-L-0001010 (isotype), KBA-L-0001037 (paratype), KBA-L-0001049 (paratype)	KBA-L-0000072, KBA-L-0002037	KBA-L-0001910, KBA-L-0001913, KBA-L-0001914, KBA-L-0001917	KBA-L-0000386, KBA-L-0002714, KBA-L-0002727, KBA-L-0002734	KBA-L-0000358, KBA-L-0000359, KBA-L-0000368, KBA-L-0002776, KBA-L-0002778, KBA-L-0002835
Ascomata sections per specimen	20	10	20	10	10
Ascomata sections per species	60	20	80	40	60

### ﻿Phylogenetic analyses

An independent phylogenetic tree for the genus *Bacidia* was produced from 84 sequences from GenBank and 12 newly-generated sequences for the new species and the new records (Table 2). All ITS sequences were aligned and edited manually using ClustalW in Bioedit v.7.2.6.1 (Hall 1999). All missing and ambiguously aligned data and phylogenetically uninformative positions were removed and phylogenetically informative regions were finally analysed in MEGA X (Stecher et al. 2020). The final alignment comprised 930 bp, in which 102 variable regions were detected. The phylogenetically informative regions were 585. Phylogenetic trees with bootstrap values were obtained in RAxML GUI 2.0 beta (Edler et al. 2019) using the Maximum Likelihood method with a rapid bootstrap with 1,000 bootstrap replications and GTR GAMMA (GTR + G4) for the substitution matrix. The posterior probabilities were obtained in BEAST 2.6.4 (Bouckaert et al. 2019) using the GTR 123454 model, as the appropriate model of nucleotide substitution produced by the Bayesian model averaging methods with bModelTest (Bouckaert and Drummond 2017), empirical base frequencies, gamma for the site heterogeneity model, four categories for gamma and a 10,000,000 Markov Chain Monte Carlo chain length with a 10,000-echo state screening and 1,000 log parameters. Then, a consensus tree was constructed in TreeAnnotator 2.6.4 (Bouckaert et al. 2019) with the first 25% discard as a burn-in, no posterior probability limit, a maximum clade credibility tree for the target tree type and median node heights. All trees were displayed in FigTree 1.4.2 (Rambaut 2014) and edited in Microsoft Paint. Overall analyses in the materials and methods were undertaken based on Lee and Hur (2020).

**Table 2.** Species list and DNA sequence information employed for phylogenetic analysis.

No.	Species	ITS	Voucher
1	*Bacidiaabsistens*	AF282085	Ekman 3223 (BG)
2	*Bacidiaalbogranulosa*	MK158340	J. Malicek 9622
3	*Bacidiaalbogranulosa*	MK158342	J. Vondrak 11888 (PRA)
4	*Bacidiaarceutina*	AF282083	Ekman 3110 (BG)
5	*Bacidiaarceutina*	JQ796851	LG DNA 579
6	*Bacidiaareolata*	MH048614	M-0182592
7	*Bacidiaauerswaldii*	AF282122	Johansson 20 (UPS)
8	*Bacidiabagliettoana*	AF282123	Ekman 3137 (BG)
9	*Bacidiabagliettoana*	MG838190	O-L-175215
10	*Bacidiabeckhausii*	AF282071	Holien 6744 (TRH)
11	*Bacidiabeckhausii*	JF714252	MSSRF Lichen Herbarium
12	*Bacidiabiatorina*	AF282079	Knutsson 94–148
13	*Bacidiacaligans*	AF282096	Johansson 21 (UPS)
14	*Bacidiacircumspecta*	MH539764	L-13006
15	*Bacidiacircumspecta*	AF282124	Ekman L1330 (LD)
16	*Bacidiacylindrophora*	MG926005	Kurokawa 1692
17	*Bacidiacylindrophora*	MG926006	Ohmura 7091 (GZU)
18	*Bacidiadiffracta*	AF282090	Wetmore 26401 (MIN)
19	*Bacidiadiffracta*	MH048620	Harris 46555-A
**20**	***Bacidiaekmaniana***	ON352611	**KBA-L-0002037**
21	*Bacidiaelongata*	MH048626	M-0182571
22	*Bacidiaelongata*	MH048629	M-0182627
23	*Bacidiafraxinea*	AF282088	Johansson 1620 (BG)
**24**	***Bacidiafriesiana***	ON352609	**KBA-L-0001910**
**25**	***Bacidiafriesiana***	ON352610	**KBA-L-0001913**
26	*Bacidiafriesiana*	MH539765	L-13159
**27**	***Bacidiafuscopallida***	ON352607	**KBA-L-0001010**
**28**	***Bacidiafuscopallida***	ON352608	**KBA-L-0001049**
29	*Bacidiafuscoviridis*	AM292665	Nordin 5058 (UPS)
30	*Bacidiagigantensis*	MT425200	MCM242
31	*Bacidiahemipolia*	AF282072	Toensberg 25091 (BG)
**32**	***Bacidiaheterochroa***	ON352606	**KBA-L-0000386**
**33**	***Bacidiaheterochroa***	ON352612	**KBA-L-0002727**
**34**	***Bacidiaheterochroa***	ON352613	**KBA-L-0002734**
35	*Bacidiahostheleoides*	AF282081	Seaward 108121
36	*Bacidiaincompta*	AF282092	Ekman 3144 (BG)
37	*Bacidiaincompta*	MG461697	KoLRI Udo-32
38	*Bacidiakurilensis*	MH048612	M-0182622
39	*Bacidiakurilensis*	MH048610	M-0182620
40	*Bacidiakurilensis*	MH048611	M-0182621
41	*Bacidialaurocerasi*	MH048609	Galanina 424
42	Bacidialaurocerasisubsp.laurocerasi	MN483106	Spribille 26334 (KLGO)
43	Bacidialaurocerasisubsp.laurocerasi	AF282078	Wetmore 74318 (MIN)
44	*Bacidialutescens*	MG925952	Ekman 3655 (BG)
45	*Bacidialutescens*	AF282082	Ekman L1161 (LD)
46	*Bacidiamedialis*	AF282102	Ekman L1193 (LD)
47	*Bacidiapolychroa*	AF282089	Knutsson 91–215
48	*Bacidiarosella*	AF282086	Ekman 3117 (BG)
49	*Bacidiarubella*	AF282087	Ekman 3021 (BG)
50	*Bacidiarubella*	HQ650644	AFTOL-ID 1793
51	*Bacidiarubella*	JQ796852	LG DNA 578
52	*Bacidiarubella*	KX132984	LIFU076–16
53	*Bacidiarubella*	MG461695	AFTOL-ID 1793
54	*Bacidiarubella*	EU266078	Hur H06122
55	*Bacidiarubella*	MH048630	M-0182581
56	*Bacidiarubella*	MK158343	J. Vondrak 12200 (PRA)
57	*Bacidiasabuletorum*	AF282069	Ekman 3091 (BG)
58	*Bacidiasachalinensis*	MH048621	M-0182619
59	*Bacidiasachalinensis*	MH048625	M-0182624
60	*Bacidiaschweinitzii*	AF282080	Wetmore 72619 (MIN)
61	*Bacidiaschweinitzii*	KX151766	Lendemer 31230A (NY)
62	*Bacidiascopulicola*	AF282084	Ekman 3106 (BG)
63	*Bacidiasigmosporae*	MW622004	P.v.d. Boom 55090
64	*Bacidiasipmanii*	JQ796853	LG DNA 361
65	*Bacidiasorediata*	KX151772	Lendemer 33787 (NY)
66	*Bacidiasorediata*	KX151775	Barton 658 (NY)
67	*Bacidiasquamulosula*	MG925955	Kalb & Kalb in Kalb, Lich. neotrop. 405
68	*Bacidiasubareolata*	MK499342	MFLU 16-0573
69	*Bacidiasubincompta*	AF282125	Ekman 3413 (BG)
70	*Bacidiasubincompta*	KX098342	WSL DF231
**71**	***Bacidiasuffusa***	ON352605	**KBA-L-0000359**
**72**	***Bacidiasuffusa***	ON352614	**KBA-L-0002776**
**73**	***Bacidiasuffusa***	ON352615	**KBA-L-0002778**
**74**	***Bacidiasuffusa***	ON352616	**KBA-L-0002835**
75	*Bacidiasuffusa*	AF282091	Wetmore 74771 (MIN)
76	*Bacidiasuffusa*	AY756456	Andersen 99 (BG)
77	*Bacidiasuffusa*	MH048615	M-0182601
78	*Bacidiasuffusa*	MH048616	M-0182593
79	*Bacidiasuffusa*	MH048617	M-0182594
80	*Bacidiasuffusa*	MH048618	M-0289887
81	*Bacidiasuffusa*	MH048619	M-0289888
82	*Bacidiasuffusa*	MW728313	LAH 36839
83	*Bacidiasuffusa*	MW788561	LAH 36838
84	*Bacidiavermifera*	AF282109	Johansson 1619 (BG)
85	*Bacidiavermifera*	KX132992	LIFU084-16 (versA)
86	*Bacidiawellingtonii*	MG925953	Ziviagina s.n.
87	*Bacidia* sp.	AY756133	KoLRI Udo-32
88	*Bacidia* sp.	KX098339	WSL DF223
89	*Bacidia* sp.	KX098340	WSL DF72
90	*Bacidia* sp.	KX098341	WSL DF80
91	*Bacidia* sp.	MG773660	Palice 19352
92	*Biatorabacidioides*	MG773663	Palice 19221
93	*Biatorabacidioides*	MG773664	Palice 19685
94	*Biatorapontica*	KF650977	C. Printzen 6114 (BG)
95	*Biatorapontica*	MK778588	J. Malicek 10212
96	*Biatoraprintzenii*	KF650978	C. Printzen 6837 (BG)
	**Overall**	**96**	

DNA sequences which were generated for the new species and the new records of *Bacidia* in this study, are presented in bold. All others were obtained from GenBank. The species names are followed by GenBank accession numbers and voucher information. ITS, internal transcribed spacer; Voucher, voucher information.

## ﻿Results and discussion

### ﻿Phylogenetic analyses

**Figure 2.**
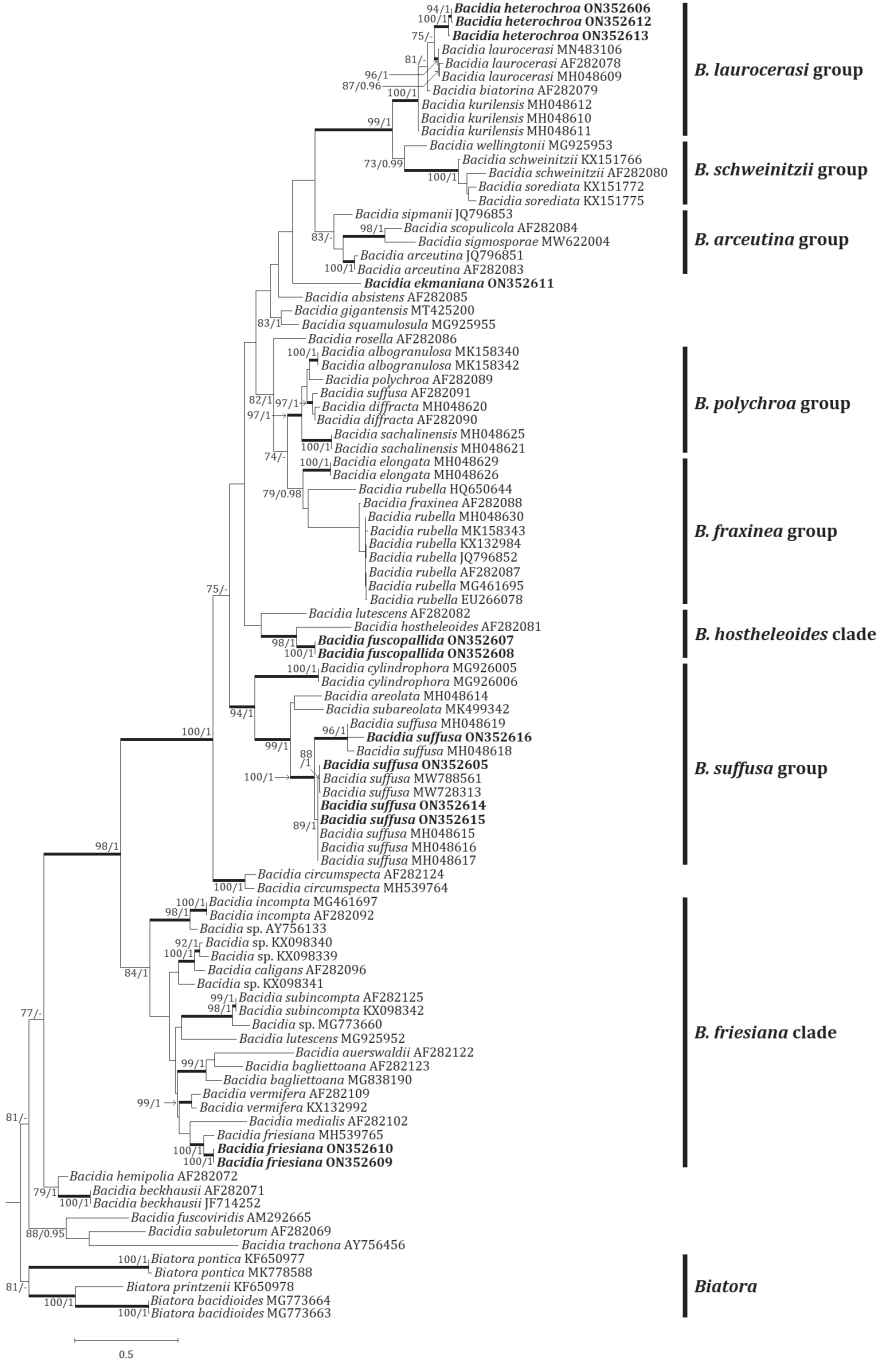
Phylogenetic relationships amongst available species in the genus *Bacidia*, based on a Maximum Likelihood analysis of the dataset of ITS sequences. The tree was rooted with the sequences of the genus *Biatora*, based on Gerasimova et al. (2018). Maximum Likelihood bootstrap values ≥ 70% and posterior probabilities ≥ 95% are shown above internal branches. Branches with bootstrap values ≥ 90% are shown as thick lines. New sequences produced in this study are presented in bold. All species names are followed by the GenBank accession numbers.

The new species is positioned in the genus *Bacidia* in the ITS tree (Fig. 2). The ITS tree describes *B.fuscopallida*, the new species, being nested with *B.hostheleoides* (Nyl.) Zahlbr., supported by a bootstrap value of 98 and a posterior probability of 1.00 for the branch. *Bacidiafuscopallida* is located in its own clade without any sequences close to it, although *B.fuscopallida* is sister to *B.hostheleoides*.

### ﻿Taxonomy

#### ﻿ Bacidia fuscopallida

Taxon classificationFungiLecanoralesRamalinaceae

B.G. Lee & T.I. Heo sp. nov.

18F5EB7C-5A14-5FD1-8B33-7562FEADCC59

843830

Fig. 3

##### Diagnosis.

*Bacidiafuscopallida* differs from *B.diffracta* by generally non-granular, olive-green thallus, pale yellow-orange apothecia without pruina, the absence of crystals in proper exciple, slightly narrower ascospores with up to 15-septation and smaller pycnidia and pycnoconidia.

##### Type.

South Korea, Gangwon Province, Gangneung, Okgye-myeon, Mt. Seokbyung, 37°34.45'N, 128°55.00'E, 271 m alt., on bark of Acerpictumvar.mono (Maxim.) Maxim. ex Franch., 17 June 2020, B.G. Lee & H.J. Lee 2020-000811, with *Porinahirsuta* Aptroot & K.H. Moon (holotype: KBA-L-0001011!); same locality, on bark of Acerpictumvar.mono, 17 June 2020, B.G. Lee & H.J. Lee 2020-000801 (isotype: KBA-L-0001001); same locality, on bark of Acerpictumvar.mono, 17 June 2020, B.G. Lee & H.J. Lee 2020-000806, with *Mikhtomiagordejevii* (Tomin) S.Y. Kondr., Kärnefelt, Elix, A. Thell, Jung Kim, A.S. Kondr. & Hur, *Straminellavaria* (Hoffm.) S.Y. Kondr., Lőkös & Farkas, *Phaeophyscialimbata* (Poelt) Kashiw., *Porinahirsuta* (isotype: KBA-L-0001006); same locality, on bark of Acerpictumvar.mono, 17 June 2020, B.G. Lee & H.J. Lee 2020-000810 (isotype: KBA-L-0001010; GenBank ON352607 for ITS); South Korea, Gangwon Province, Gangneung, Okgye-myeon, Mt. Seokbyung, 37°34.39' N, 128°55.01'E, 349 m alt., on bark of *Quercusmongolica* Fisch. ex Ledeb., 17 June 2020, B.G. Lee & H.J. Lee 2020-000837, with *Opeltiaflavorubescens* (Huds.) S.Y. Kondr. & Hur (paratype: KBA-L-0001037); South Korea, Gangwon Province, Gangneung, Okgye-myeon, Mt. Seokbyung, 37°34.28'N, 128°54.88'E, 438 m alt., on bark of *Acertriflorum* Kom., 17 June 2020, B.G. Lee & H.J. Lee 2020-000849, with *Biatorapacifica* Printzen, Tønsberg & G. Thor (paratype: KBA-L-0001049; GenBank ON352608 for ITS).

Thallus corticolous, crustose, areoles in young stage and soon coarsely continuous or warted on aging, often overlapping for each other, rarely granular, thin when not overlapping, olivish-green, margin indeterminate, 40–90 μm thick; cortex indistinct, hyaline, up to 5 μm thick; medulla a little shown as mycelia below algal layer; photobiont chlorococcoid, cells globose to subglobose, 5–15 μm thick, algal layer composing most part of thallus, 35–80 μm thick. Prothallus indistinct or whitish-grey and endosubstratal when present.

Apothecia numerous, solitary, marginate and flat in young stage and seeming immarginate and convex on aging (consistently marginate and flat on bark of *Acertriflorum*), 0.1–0.7 mm diam. (mean = 0.33; SD = 0.14; n = 105). Pruina absent. Disc biatorine, thalline exciple absent, pale yellow to pale orange in young stage and slightly more blackish generally around margin when mature (much more blackish on bark of *A.triflorum* and *Q.mongolica* from young stage). Proper exciple 65–80 μm wide laterally (SD = 5.7; n = 15), with radiating hyphae of 1–2.5 μm wide (SD = 0.5; n = 10) and outermost cell 2.5–4 μm wide (SD = 0.6; n = 10), hyaline to pale yellow around rim, but darker downwards (pale yellow to pale brown) and the dark colour extending to hypothecium. Epihymenium hyaline, with a little pigment of pale yellow to pale olive-brown locally, smooth and not granular, ca. 5 μm high. Hymenium hyaline, 70–100 μm high (SD = 8.9; n = 10). Hypothecium clearly pigmented, pale orange-brown to brown, prosoplectenchymatous (irregularly arranged), 70–130 μm high (SD = 18.9; n = 10). Crystals absent or a little present in upper hypothecium. Oil droplets absent. Paraphyses simple, rarely branched at tips, 1–1.5 μm wide, tips not or little swollen, not pigmented, 1.5–2 μm wide. Asci cylindrical to narrowly clavate, 8-spored, 49–72 × 11–14 μm (SD = 7.3 (L), 0.9 (W); n = 11). Ascospores 3- to 15-septate, acicular to filiform, 24–69 × 2–3.5 μm (mean = 52.8 × 2.6 μm; SD = 8.7 (L), 0.6 (W); L/W ratio = 3.8–30.5, ratio mean = 17.6, ratio SD = 5.0; n = 104). Pycnidia black, immersed and upper half only shown, globose, 60–65 μm high and 55–75 μm wide (SD = 2.4 (H), 8.2 (W); n = 5), with brownish wall, K–. Pycnoconidia hyaline, filiform, curved or almost straight, 6–17 × 0.3–0.5 μm (mean = 10.4 × 0.5 μm; SD = 2.9 (L), 0.1 (W); n = 53).

##### Chemistry.

Thallus K– or K+ slightly yellow, KC–, C–, Pd–, UV–. Epihymenium K+ purple extending to outermost layers of proper exciple, C–. No lichen substance was detected by TLC.

##### Distribution and ecology.

The species occurs on barks of Acerpictumvar.mono, *A.triflorum* and *Quercusmongolica*. The species is currently known from the type collections.

##### Etymology.

The species epithet indicates the pale brown colour of the lichen’s apothecia.

##### Notes.

The new species is similar to *B.diffracta* and *B.polychroa* (Th. Fr.) Körb. in having colourless epihymenium with pale orange-brown pigment and K+ purple reaction, distinctly pigmented hypothecium with yellow, orange or brown, long ascospores generally with L/W ratio over 10 amongst corticolous species. However, *B.diffracta* differs from the new species by granular thallus, darker and larger apothecia with pruina, proper exciple with radiating clusters of minute crystals, slightly wider ascospores with up to 11-septation and larger pycnidia and pynoconidia (Ekman 1996) (Table 3).

**Table 3.** Comparison of the new species with close species in the genus *Bacidia*.

Species	*Bacidiafuscopallida*	*Bacidiadiffracta*	*Bacidiahostheleoides*	*Bacidiapolychroa*	*Bacidiapurpurans*
Thallus growth form	warted, rarely granular	finely granular	wrinkled or granular to subsquamulose	finely wrinkled to warted, sometimes areolate	areolate
Thallus colour	olivish-green	pale grey, green-grey, yellow-grey to grey	pale grey to pale green-grey	white to grey or yellow-grey	pale grey-green to dark green
Prothallus	white-grey around margin, endosubstratal	white-pale grey between granules, endosubstratal	absent	–	white, arachnoid
Apothecia (mm in diam.)	0.1–0.7	0.5–1.1	0.5–0.8	0.4–1.2	–
Disc colour	pale yellow to pale orange (young); more blackish (old)	brown-orange to dark brown	brown-orange	brown-orange to dark brown	dark purple-brown to brown
Pruina	absent	white	absent	white	absent
Crystals in proper exciple	absent	radiating clusters of minute crystals	absent	with or without radiating clusters of minute crystals	absent
Crystals in hymenium	small crystals at bottom	–	–	–	absent
Epihymenium colour	colourless with pale yellow-brown pigment	colourless with pale orange-brown pigment	very pale orange	colourless with brown-orange pigment	greyish
Hymenium height (μm)	70–100	70–100	ca. 60	55–100	ca. 100
Hypothecium colour	pale orange-brown to brown	pale brown to orange-brown	very pale orange	brown-orange to dark brown	orange-brown
Hypothecium height (μm)	70–130	–	–	–	ca. 60
Ascospore (μm)	24–69 × 2–3.5	32–69 × 1.9–4.1	16–25 × 2.9–5	31–74 × 1.9–5	50–75 × 2–4
Ascospore L/W ratio	4–31	9–27	4–9	7–30	–
Ascospore septation	3–15	3–11	3–5	2–15	3–15
Pycnidia (μm)	55–75	150	50–100	100–170	150–200
Pycnoconidia	6–17 × 0.3–0.5	10–15 × 0.5–0.6	①10–14 × 0.5 ②6–9 × 1.6–2	10–17 × 0.6–0.8	20–25 × 0.8
Substance	absent	atranorin, (trace of zeorin)	absent	(trace of atranorin)	atranorin
Reference	this study	Ekman (1996)	Ekman (1996)	Ekman (1996); Smith et al. (2009)	Lendemer et al. (2016)

The morphological and chemical characteristics of several species close to the new species are referenced from the previous literature. All information on the new species is produced from type specimens (KBA-L-0001010, KBA-L-0001011 and KBA-L-0001049) in this study.

The new species is more similar to *B.polychroa* in having coarsely continuous or warted thallus. However, *B.polychroa* differs from the new species by greyish thallus, darker and larger apothecia often with pruina, proper exciple often with radiating clusters of minute crystals, wider ascospores and larger pycnidia and pycnoconidia (Ekman 1996; Smith et al. 2009) (Table 3).

The new species is quite similar to *B.purpurans* R. C. Harris, Ladd & Lendemer in having greenish thallus with areoles and K+ purple reaction in epihymenium. However, *B.purpurans* differs from the new species by arachnoid prothallus, darker apothecia, green excipular rim adjacent to epihymenium, greyish epihymenium, shorter hypothecium, absence of crystals, larger ascospores and larger pycnidia and pycnoconidia (Lendemer et al. 2016) (Table 3).

**Figure 3.**
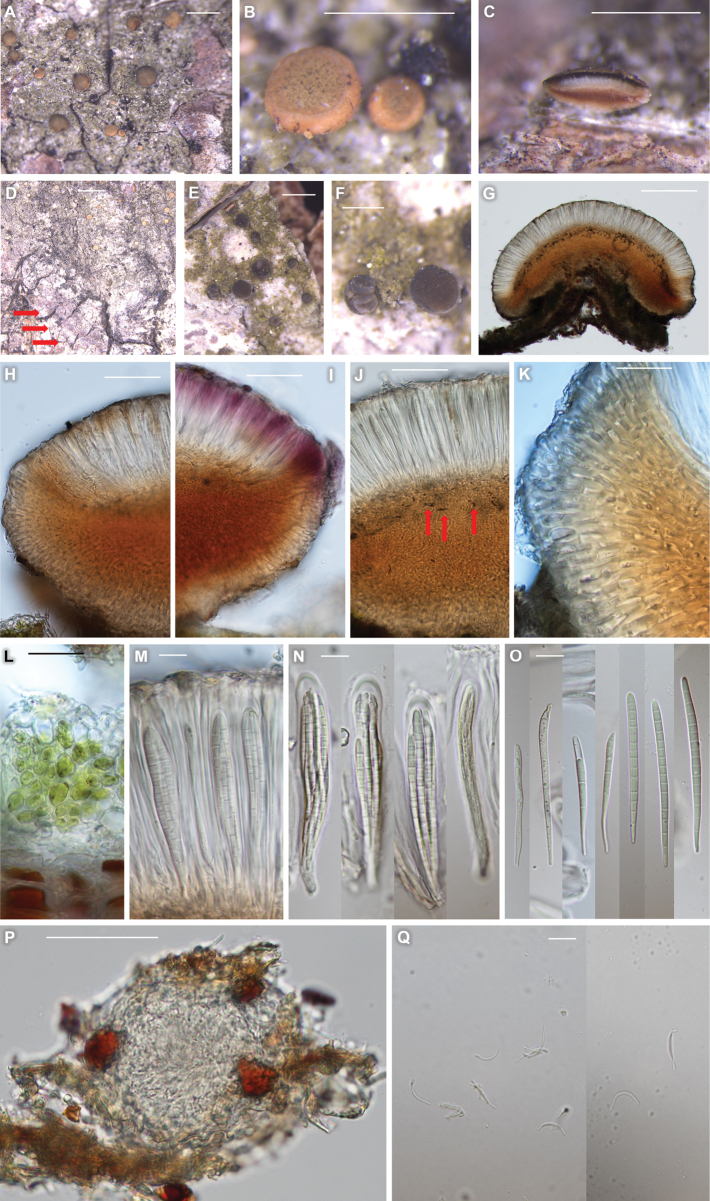
*Bacidiafuscopallida* (KBA-L-0001011, holotype for **A–D, G–O**KBA-L-0001049 for **E, F**KBA-L-0001010 for **P, Q**) in morphology **A, B** habitus and apothecia on bark of Acerpictumvar.mono. Olive-green thallus and pale yellow-orange apothecia **C** vertical section of apothecia **D** prothallus present around margin of habitus (red arrows) **E, F** habitus and apothecia growing on bark of *Acertriflorum***G** apothecial section **H** epihymenium colourless or a little pigmented **I** epihymenium K+ purple **J** small crystals (red arrows) present in upper hypothecium **K** proper exciple pigmented with pale or colourless margin. Radiating hyphae wider to margin **L** photobiont composing most part of thallus **M, N** asci cylindrical to narrowly clavate. Ascospores not twisted in ascus **O** ascospores acicular to filiform up to 15-septate **P** pycnidia globose with brown wall **Q** pycnoconidia curved or almost straight. Scale bars: 1 mm (**A, E**); 500 μm (**B, C, F**); 2 mm (**D**); 200 μm (**G**); 50 μm (**H–J, P**); 20 μm (**K, L**); 10 μm (**M–O, Q**).

The new species can be compared with *B.hostheleoides* in sharing non-pruinose apothecia and proper exciple without crystals. However, *B.hostheleoides* differs from the new species by greyish thallus, absence of prothallus, shorter hymenium, paler hypothecium and shorter ascospores with a few septa (Ekman 1996) (Table 3).

#### ﻿ Bacidia ekmaniana

Taxon classificationFungiLecanoralesRamalinaceae

R. C. Harris, Ladd & Lendemer, The Bryologist 119 (2): 154 (2016)

76946801-3ED4-514A-BB6B-1F6B978B6F72

Fig. 4

##### Description.

Thallus corticolous, crustose, somewhat granular when young and smoother when mature, grey, greenish-grey to pale grey, margin indeterminate. Prothallus generally not detected or whitish-grey when present.

Apothecia consistently flat or slightly convex when mature, marginate, without pruina, 0.4–1.4 mm diam. (mean = 0.75, SD = 0.23, n = 104). Disc biatorine, without thalline exciple, pale straw, light brown to brown, with a distinct proper margin which is smooth to rugose and becoming thinner but still distinct when mature. Proper exciple pale brown to red-brown, paler or colourless around rim and thicker downwards, 80–120 μm wide laterally. Epihymenium hyaline, smooth but not granular, ca. 5 μm high. Hymenium hyaline, 80–140 μm high. Hypothecium red-brown, prosoplectenchymatous (irregularly arranged), 120–250 μm high. Small crystals present a little in hypothecium, dissolving in K. Oil droplets absent. Asci narrowly clavate, 8-spored, 70–105 × 8–12 μm (n = 5). Ascospores acicular to filiform, cells near head sometimes irregularly swollen, 3- to 9-septate, 52–71 × 2–4.5 μm (n = 15). Pycnidia not detected.

**Figure 4.**
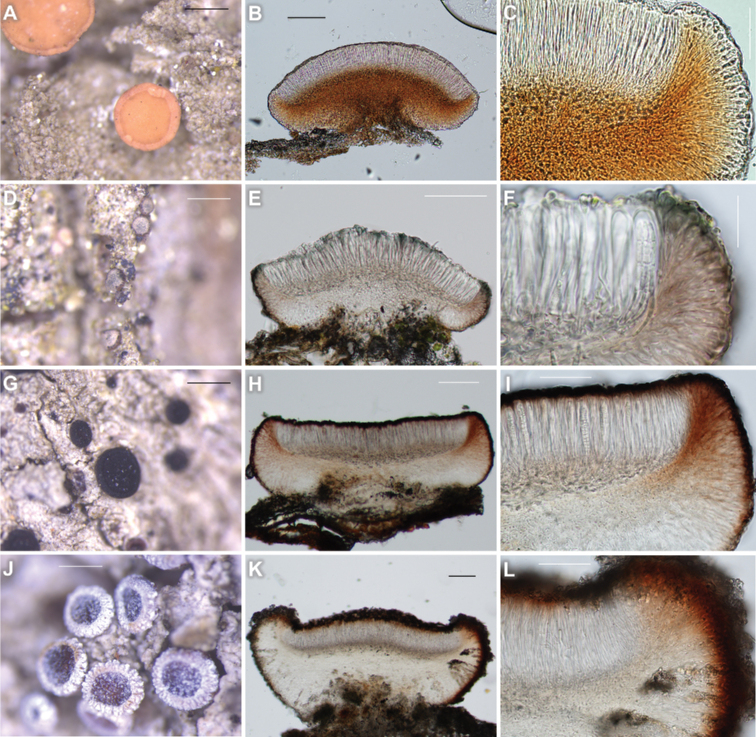
Four new records of *B.ekmaniana* (KBA-L-0000412 for **A–C**), *B.friesiana* (KBA-L-0001914 for **D–F**), *B.heterochroa* (KBA-L-0000386 for **G–I**) and *B.suffusa* (KBA-L-0000359 for **J–L**) in morphology **A** habitus and apothecia. Granular thallus with green-grey pigment and straw-coloured apothecia **B–C** apothecial section with colourless epihymenium, red-brown hypothecium, and pale excipulum **D** habitus and apothecia. Thallus pale grey with slightly brownish pigment and pale pink apothecia **E, F** apothecial section with greenish epihymenium **G** habitus and apothecia. Thallus pale yellowish-grey and black apothecia with red pigment **H, I** apothecial section and proper exciple with dark margin **J** habitus and apothecia. Thallus whitish pale grey and pruinose apothecia **K, L** apothecial section with radiating clusters of crystals, which produce pruina on surface. Scale bars: 500 μm (**A, D, G, J**); 100 μm (**B, E, H, K**); 50 μm (**C, I, L**); 20 μm (**F**).

##### Chemistry.

Thallus K–, C–. Apothecial section K–, C–. No lichen substance was detected by TLC.

##### Notes.

*Bacidiaekmaniana* is easily confused with *B.schweinitzii* under the microscope, as well as in the field because both species often share their habitat and the habiti of both species look similar particularly when the ascomata of the latter are paler. Both species are often detected from one specimen under the microscope and those were frequently regarded as one species, i.e. *B.schweinitzii*. Generally, however, *B.ekmaniana* differs from the latter by paler ascomata. *Bacidiaekmaniana* has brown but not black apothecia when mature (Lendemer et al. 2016). *Bacidiaekmaniana* differs from the latter by colourless epihymenium and paler hypothecium as well.

*Bacidiaekmaniana* is more similar to *B.arceutina* than *B.schweinitzii* in morphology in having pale ascomata. However, *B.ekmaniana* differs from *B.arceutina* by the colourless to pale excipular rim, colourless epihymenium and wider ascospores with more septation (Ekman 1996; also see the key couplet 23). *Bacidiaekmaniana* is new to Asia and this is the second record after North America (Lendemer et al. 2016). *Bacidiaekmaniana* is supposed to occur widespread throughout the world as the species was assumed to be *B.schweinitzii* in the past. Phylogenetic analysis resulted in *B.ekmaniana* being located in its own clade in the genus *Bacidia* (Fig. 2).

##### Specimens examined.

South Korea, North Gyeongsang Province, Bonghwa, Chunyang-myeon, Mt. Munsu, 36°59.28'N, 128°48.17'E, 1,058 m alt., on bark of *Quercusmongolica*, 29 August 2019, B.G. Lee 2019-000072 (KBA-L-0000072); South Korea, South Jeolla Province, Gokseong, Jukgok-myeon, Taeansa Temple, 35°08.06'N, 127°23.26'E, 297 m alt., on bark of *Salixpierotii* Miq., 25 May 2020, B.G. Lee 2020-000212, with *Bacidiaschweinitzii* (KBA-L-0000412); same locality, on bark of *Salixpierotii*, 25 May 2020, B.G. Lee 2020-000227, with *Bacidiaschweinitzii*, *Coenogoniumpineti* (Ach.) Lücking & Lumbsch, *Phaeophysciarubropulchra* (Degel.) Moberg, *Porinamelanops* Malme (KBA-L-0000427); same locality, on bark of *Idesiapolycarpa* Maxim., 25 May 2020, B.G. Lee 2020-000231, with *Bacidiaschweinitzii*, Porinaaff.melanops (KBA-L-0000431); same locality, on bark of *Idesiapolycarpa*, 25 May 2020, B.G. Lee 2020-000232 (KBA-L-0000432); same locality, on bark of *Taxicodendronvernicifluum* (Stokes) F. A. Barkley, 25 May 2020, B.G. Lee 2020-000233, with Biatoraaff.pacifica, *Lecidea* sp., *Phaeophysciarubropulchra*, *Rinodina* sp., *Traponoravarians* (Ach.) J. Kalb & Kalb (KBA-L-0000433); South Korea, North Gyeongsang Province, Bonghwa, Chunyang-myeon, Mt. Okseok, 37°00.91'N, 128°46.65'E, 1,085 m alt., on bark of *Quercusmongolica*, 15 September 2020, B.G. Lee & H.J. Lee 2020-001159, with *Anisomeridiumpolypori* (Ellis & Everh.) M.E. Barr, *Bacidiaschweinitzii*, *Rinodina* sp. (KBA-L-0001359); same locality, on bark of *Quercusmongolica*, 15 September 2020, B.G. Lee & H.J. Lee 2020-001162, with *Rinodina* sp. (KBA-L-0001362); South Korea, North Jeolla Province, Jangsu, Mt. Youngchui, 35°38.59'N, 127°37.00'E, 907 m alt., on bark of *Carpinustschonoskii* Maxim., 08 June 2021, B.G. Lee & H.J. Lee 2021-000563, with *Lecanoramegalocheila* (Hue) H. Miyaw., *Rinodinaorientalis* Sheard (KBA-L-0002035); same locality, on bark of *Carpinustschonoskii*, 08 June 2021, B.G. Lee & H.J. Lee 2021-000565, with *Arthoniaapatetica* (A. Massal.) Th. Fr., *Lecidellaeuphorea* (Flörke) Kremp. (KBA-L-0002037; GenBank ON352611 for ITS); same locality, on bark of *Carpinustschonoskii*, 08 June 2021, B.G. Lee & H.J. Lee 2021-000569, with *Anisomeridiumpolypori*, *Lecidellaeuphorea*, *Rinodinaorientalis*, *Scoliciosporum* sp. (KBA-L-0002041); same locality, on bark of *Carpinustschonoskii*, 08 June 2021, B.G. Lee & H.J. Lee 2021-000573, with *Arthoniaapatetica*, Lecanoraaff.imshaugii Brodo, *Lecidellaeuphorea*, *Porinahirsuta* (KBA-L-0002045); South Korea, North Jeolla Province, Jangsu, Mt. Jangan, 35°38.58'N, 127°36.96'E, 925 m alt., on bark of *Carpinustschonoskii*, 09 June 2021, B.G. Lee & H.J. Lee 2021-000759 (KBA-L-0002231); same locality, on bark of *Carpinustschonoskii*, 09 June 2021, B.G. Lee & H.J. Lee 2021-000760, with *Phaeophysciaadiastola* (Essl.) Essl., *Porinahirsuta*, *Rinodinaorientalis*, *Scoliciosporumchlorococcum* (Graewe ex Stenh.) Vězda (KBA-L-0002232); same locality, on bark of *Carpinustschonoskii*, 09 June 2021, B.G. Lee & H.J. Lee 2021-000766, with *Lecania* sp., *Phaeophyscia* sp., *Rinodinaorientalis* (KBA-L-0002238); South Korea, North Jeolla Province, Jangsu, Mt. Baegun, 35°36.76'N, 127°36.85'E, 661 m alt., on bark of *Cornuswalteri* Wangerin, 10 June 2021, B.G. Lee & H.J. Lee 2021-000926 (KBA-L-0002398); same locality, on bark of *Cornuswalteri*, 10 June 2021, B.G. Lee & H.J. Lee 2021-000927 (KBA-L-0002399); same locality, on bark of *Cornuswalteri*, 10 June 2021, B.G. Lee & H.J. Lee 2021-000928 (KBA-L-0002400); same locality, on bark of *Cornuswalteri*, 10 June 2021, B.G. Lee & H.J. Lee 2021-000929, with *Phaeophysciaadiastola* (KBA-L-0002401); same locality, on bark of *Cornuswalteri*, 10 June 2021, B.G. Lee & H.J. Lee 2021-000930, with *Phaeophysciarubropulchra* (KBA-L-0002402); same locality, on bark of *Cornuswalteri*, 10 June 2021, B.G. Lee & H.J. Lee 2021-000931, with *Lecanora* sp., *Phaeophysciaadiastola* (KBA-L-0002403); same locality, on bark of *Cornuswalteri*, 10 June 2021, B.G. Lee & H.J. Lee 2021-000932 (KBA-L-0002404).

#### ﻿ Bacidia friesiana

Taxon classificationFungiLecanoralesRamalinaceae

(Hepp) Körb., Parerga lichenol. (Breslau) 2: 133 (1860) [1865]

39A1D2D0-641D-5B56-A680-EE4D2E7BC165

Fig. 4

##### Description.

Thallus corticolous, crustose, thin, little developed or indistinct, generally not continuous, minutely granular with contiguous granules when developed, pale grey with slightly brownish colour, margin indeterminate. Prothallus not detected.

Apothecia consistently flat or convex when mature, marginate, without pruina, 0.1–0.5 mm diam. (mean = 0.23, SD = 0.07, n = 107). Disc biatorine, without thalline exciple, pale pink to pale yellow when young and darker (particularly around margin) when mature. Proper exciple hyaline with or without pale brown pigment, the pigment slightly thicker close to hymenium or excipular rim, 40–50 μm wide laterally. Epihymenium bluish-green, ca. 5 μm high. Hymenium hyaline, 40–45 μm high. Hypothecium hyaline, 50–60 μm high; upper hypothecium paraplectenchymatous (globular to angular), lower hypothecium prosoplectenchymatous (periclinally or irregularly arranged). Crystals or oil droplets absent. Asci narrowly clavate, 8-spored, 39–41 × 10–12 μm (n = 3). Ascospores acicular to filiform, 3- or 7-septate, 28–38 × 1.5–2.5 μm (n = 14). Pycnidia not detected.

##### Chemistry.

Epihymenium K–, C–. Hymenium K– or a few undeveloped asci K+ purplish. No lichen substance was detected by TLC.

##### Notes.

*Bacidiafriesiana* is similar to *B.circumspecta* (Norrl. & Nyl.) Malme and *B.igniarii* (Nyl.) Oxner (syn. *Scutulaigniarii* (Nyl.) S. Ekman) in having epihymenium with green pigments, proper exciple without crystals and dark hypothecium amongst corticolous species. However, *B.friesiana* differs from the latter two by the excluded margin of apothecia and acicular ascospores. The latter species have a permanent margin of apothecia and bacilliform or clavate ascospores (Ekman 1996).

Phylogenetic analysis resulted in *B.friesiana* of Korea (ON352609 and ON352610) being nested with the sequences of Russia (MH539765), supported by a bootstrap value of 100 and a posterior probability of 1.00 for the branch (Fig. 2). *Bacidiafriesiana* was previously reported from Europe, North America and Russian Far East (Smith et al. 2009; Gerasimova et al. 2018). This is a new record to Korea.

##### Specimens examined.

South Korea, Gangwon Province, Yanggu, Nam-myeon, Dumu-ri, nearby a forested wetland, 38°02.12'N, 128°05.14'E, 421 m alt., on bark of *Salixpierotii*, 28 April 2020, B.G. Lee 2020-000164, with *Mikhtomiagordejevii*, *Candelariaconcolor* (Dicks.) Arnold, *Phaeophysciaadiastola*, Porinacf.melanops, Rinodinacf.subminuta (KBA-L-0000364); South Korea, Gyeonggi Province, Yangpyeong, Cheongun-myeon, Dowon-ri, a forested wetland, 37°32.55'N, 127°48.60'E, 443 m alt., on bark of *Salixpierotii*, 31 May 2021, B.G. Lee & H.J. Lee 2021-000438, with *Lecidellaeuphorea*, *Phaeophysciaadiastola*, *Rinodinaorientalis* (KBA-L-0001910; GenBank ON352609 for ITS); same locality, on bark of *Araliaelata* (Miq.) Seem., 31 May 2021, B.G. Lee & H.J. Lee 2021-000440, with *Lecidellaeuphorea*, *Phaeophysciaadiastola*, *Traponoravarians* (KBA-L-0001912); same locality, on bark of *Araliaelata*, 31 May 2021, B.G. Lee & H.J. Lee 2021-000441, with *Hyperphysciaadglutinata* (Flörke) H. Mayrhofer & Poelt, *Rinodinaorientalis* (KBA-L-0001913; GenBank ON352610 for ITS); same locality, on bark of *Araliaelata*, 31 May 2021, B.G. Lee & H.J. Lee 2021-000442, with *Rinodinaorientalis*, *Traponoravarians* (KBA-L-0001914); same locality, on bark of *Araliaelata*, 31 May 2021, B.G. Lee & H.J. Lee 2021-000443, with *Hyperphysciaadglutinata*, *Rinodinaorientalis*, *Traponoravarians* (KBA-L-0001915); same locality, on bark of *Araliaelata*, 31 May 2021, B.G. Lee & H.J. Lee 2021-000444, with *Phaeophysciaadiastola*, *P.rubropulchra*, *Rinodinaorientalis* (KBA-L-0001916); same locality, on bark of *Araliaelata*, 31 May 2021, B.G. Lee & H.J. Lee 2021-000445 (KBA-L-0001917).

#### ﻿ Bacidia heterochroa

Taxon classificationFungiLecanoralesRamalinaceae

(Müll. Arg.) Zahlbr., Cat. Lich. Univers. 4: 204 (1926) [1927]

ACCF9C98-EE5A-5D83-8A10-71E9ED3D598A

Fig. 4

##### Description.

Thallus corticolous, crustose, continuous, wrinkled, or warted, pale yellowish-grey, margin indeterminate or determinate. Prothallus generally not present or locally present as blackish bordering a different lichen.

Apothecia flat, marginate, without pruina, 0.2–0.6 mm diam. (mean = 0.33, SD = 0.11, n = 72). Disc lecideine, without thalline exciple, blackish or reddish-black. Proper exciple hyaline with pale brown pigment dispersed, pigment slightly thicker close to hymenium, 80–100 μm wide laterally. Epihymenium brown to dark brown, ca. 10 μm high. Hymenium hyaline, 80–95 μm high. Hypothecium hyaline, 80–120 μm high, with a little pale yellow pigment. Crystals or oil droplets absent. Asci narrowly clavate to cylindrical, 8-spored, 42–48 × 12–13 μm (n = 3). Ascospores acicular to filiform, 9- or 10-septate, 36–67 × 2.5–4 μm (n = 11). Pycnidia not detected.

##### Chemistry.

Epihymenium K+ purple or intensifying, extending to excipular rim. No lichen substance was detected by TLC.

##### Notes.

*Bacidiaheterochroa* is the most similar to *B.laurocerasi* (Delise ex Duby) Zahlbr. in having smooth thallus without granules, absence of crystals in exciple, epihymenium without green pigments, pale to colourless hypothecium, K+ purple in apothecial section and narrow ascospores less than 4 μm wide amongst corticolous species. However, *B.heterochroa* differs from *B.laurocerasi* by distinctly brown-pigmented paraphysial tips, less than 16-septate ascospores which are shorter but wider (less than 80 μm long but over 3.5 μm wide) and substrate preference to deciduous trees or shrubs (Ekman 1996; Brodo 2016; also see the key couplet 21).

Phylogenetic analysis resulted in *B.heterochroa* of Korea (ON352606, ON352612 and ON352613) being nested in a sister clade to *B.laurocerasi*, supported by a bootstrap value of 75 without a posterior probability as the Maximum Likelihood analysis did not match with the Bayesian Inference for the clade. The sequences of *B.heterochroa* were not compared with previous records due to the lack of data (Fig. 2). *Bacidiaheterochroa* was previously reported from Thailand in Asia (Aptroot et al. 2007) and this is a new record to northeastern Asia.

##### Specimens examined.

South Korea, Gangwon Province, Yanggu, Nam-myeon, Dumu-ri, a forested wetland, 38°02.12'N, 128°05.14'E, 421 m alt., on bark of *Salixkoriyanagi* Kimura ex Goerz, 28 April 2020, B.G. Lee 2020-000186 (KBA-L-0000386; GenBank ON352606 for ITS); South Korea, South Jeolla Province, Damyang, Changpyeong-myeon, Oedong-ri, a forested wetland, 35°12.00'N, 127°00.88'E, 338 m alt., on bark of *Fraxinusrhynchophylla* Hance, 12 May 2021, B.G. Lee & D.Y. Kim 2021-000214 (KBA-L-0001686); South Korea, Gangwon Province, Jeongseon, Imgye-myeon, Gamok-ri, a forested wetland, 37°32.47'N, 128°57.72'E, 760 m alt., on bark of Acertartaricumsubsp.ginnala (Maxim.) Wesm., 17 June 2021, B.G. Lee & H.J. Lee 2021-001241, with *Lecanorachionocarpa* Hue (KBA-L-0002713); same locality, on bark of Acertartaricumsubsp.ginnala, 17 June 2021, B.G. Lee & H.J. Lee 2021-001242, with *Phaeophysciaadiastola* (KBA-L-0002714); same locality, on bark of Acertartaricumsubsp.ginnala, 17 June 2021, B.G. Lee & H.J. Lee 2021-001255, with *Opeltiaflavorubescens*, *Phaeophysciaadiastola* (KBA-L-0002727; GenBank ON352612 for ITS); same locality, on bark of Acertartaricumsubsp.ginnala, 17 June 2021, B.G. Lee & H.J. Lee 2021-001257, with *Hyperphysciaadglutinata*, *Lecidellaeuphorea* (KBA-L-0002729); same locality, on bark of Acertartaricumsubsp.ginnala, 17 June 2021, B.G. Lee & H.J. Lee 2021-001262, with *Lecidellaeuphorea*, *Phaeophysciaadiastola*, *Rinodinaorientalis* (KBA-L-0002734; GenBank ON352613 for ITS); same locality, on bark of Acertartaricumsubsp.ginnala, 17 June 2021, B.G. Lee & H.J. Lee 2021-001263, with *Opeltiaflavorubescens*, *Phaeophysciaadiastola*, *Rinodinaorientalis* (KBA-L-0002735); same locality, on bark of Acertartaricumsubsp.ginnala, 17 June 2021, B.G. Lee & H.J. Lee 2021-001267, with *Lecidellaeuphorea*, *Porinahirsuta*, *Rinodinaorientalis*, *Straminellavaria* (KBA-L-0002739); same locality, on bark of Acertartaricumsubsp.ginnala, 17 June 2021, B.G. Lee & H.J. Lee 2021-001269, with *Lecidellaeuphorea*, *Opeltiaflavorubescens*, *Phaeophysciarubropulchra*, *Rinodinaorientalis* (KBA-L-0002741).

#### ﻿ Bacidia suffusa

Taxon classificationFungiLecanoralesRamalinaceae

(Fr.) A. Schneid., Guide Study Lich.: 110 (1898)

F74E4A8A-FF9F-5AAB-A04D-4605B87A005A

Fig. 4

##### Description.

Thallus corticolous, crustose, continuous, wrinkled, warted or subsquamulose, often granular locally, whitish pale grey. Prothallus generally not present or present as dark brown to black between different colonies.

Apothecia flat, marginate, with a little or heavy white pruina, generally more pruinose at margin, 0.3–1.7 mm diam. (mean = 0.75, SD = 0.28, n = 116). Disc lecideine, without thalline exciple, brown to dark brown. Proper exciple with radiating clusters of crystals produced around hypothecium and expanding to excipular rim and finally shown as pruina on surface, hyaline downwards but brown around rim, the brown concolorous or slightly paler to epihymenium, 80–100 μm wide laterally. Epihymenium brown to dark brown, ca. 10 μm high, with pruina (ca. 10 μm high) on surface. Hymenium hyaline, 70–80 μm high. Hypothecium hyaline, 80–100 μm high. Other small crystals present a few in upper hypothecium. Oil droplets absent. Asci cylindrical, 8-spored, 65–75 × 10–16 μm (n = 7). Ascospores acicular to filiform, up to 13-septate, 45–70 × 2.5–4.5 μm (n = 10). Pycnidia not detected.

##### Chemistry.

Thallus K+ yellow, KC–, C–, Pd–, UV–. Epihymenium K–. Atranorin was detected by TLC.

##### Notes.

*Bacidiasuffusa* is the most similar to *B.russeola* (Kremp.) Zahlbr. in having dark apothecia, generally colourless epihymenium without green pigment, long ascospores with the L/W ratio over 11, pale or colourless hypothecium and K+ purple reaction on epihymenium and nearby excipular rim amongst corticolous species. However, *B.suffusa* differs from *B.russeola* by the presence of pruina on the disc and in proper exciple as radiating clusters of crystals and more than 10-septate ascospores (Ekman 1996).

Phylogenetic analysis resulted in *B.suffusa* of Korea (ON352605, ON352614, ON352615 and ON352616) being nested in a sister clade of the sequences of Pakistan (MW728313 and MW788561), Russia (MH048615, MH048616 and MH048617) or U.S.A. (MH048618 and MH048619). The molecular data of Korea converged into the previous data of *B.suffusa*, supported by a bootstrap value of 100 and a posterior probability of 1.00 for the branch (Fig. 2). *Bacidiasuffusa* was previously detected from North America, North Caucasus, Russian Far East and Pakistan, but rare or absent in Europe (Otte 2007; Gerasimova et al. 2018, 2021; Adrees et al. 2022). This is a new record to Korea.

##### Specimens examined.

South Korea, Gangwon Province, Yanggu, Nam-myeon, Dumu-ri, a forested wetland, 38°02.12'N, 128°05.14'E, 421 m alt., on bark of *Salixpierotii* Miq., 28 April 2020, B.G. Lee 2020-000158 (KBA-L-0000358); same locality, on bark of *Salixpierotii*, 28 April 2020, B.G. Lee 2020-000159 (KBA-L-0000359; GenBank ON352605 for ITS); same locality, on bark of *Salixpierotii*, 28 April 2020, B.G. Lee 2020-000168, with *Candelariaconcolor*, *Phaeophysciaadiastola*, *Phaeophysciahirtuosa* (Kremp.) Essl. (KBA-L-0000368); South Korea, Gangwon Province, Gangneung, Okgye-myeon, Mt. Seokbyung, 37°34.45'N, 128°55.01'E, 271 m alt., on bark of Acerpictumvar.mono, 17 June 2020, B.G. Lee & H.J. Lee 2020-000799 (KBA-L-0000999); South Korea, Gangwon Province, Jeongseon, Imgye-myeon, Gamok-ri, a forested wetland, 37°32.47'N, 128°57.72'E, 760 m alt., on bark of *Fraxinuschiisanensis* Nakai, 17 June 2021, B.G. Lee & H.J. Lee 2021-001304, with *Normandinapulchella* (Borrer) Nyl., *Phaeophyscia* sp. (KBA-L-0002776; GenBank ON352614 for ITS); same locality, on bark of *Fraxinuschiisanensis*, 17 June 2021, B.G. Lee & H.J. Lee 2021-001305, with *Anisomeridiumpolypori*, *Normandinapulchella*, *Phaeophyscia* sp., *Porinahirsuta* (KBA-L-0002777); same locality, on bark of *Fraxinuschiisanensis*, 17 June 2021, B.G. Lee & H.J. Lee 2021-001306, with *Normandinapulchella*, *Opeltiaflavorubescens*, *Phaeophysciaadiastola* (Essl.) Essl. (KBA-L-0002778; GenBank ON352615 for ITS); same locality, on bark of *Fraxinuschiisanensis*, 17 June 2021, B.G. Lee & H.J. Lee 2021-001308, with *Phaeophysciaadiastola* (KBA-L-0002780); same locality, on bark of *Fraxinuschiisanensis*, 17 June 2021, B.G. Lee & H.J. Lee 2021-001320, with *Opeltiaflavorubescens* (KBA-L-0002792); same locality, on bark of Acertartaricumsubsp.ginnala, 17 June 2021, B.G. Lee & H.J. Lee 2021-001363 (KBA-L-0002835; GenBank ON352616 for ITS).

### ﻿Key to the species of *Bacidia* in Korea (19 taxa)

The key is composed of all 19 species in the genus *Bacidia* of Korea, including synonyms in *Bacidina* and *Toniniopsis* species.

**Table:** 

1	Epihymenium with green pigment	**2**
–	Epihymenium colourless, yellow-brown, brown to dark brown, but without green pigment	**5**
2	Proper exciple with radiating clusters of coarse crystals (up to 7 μm wide); hymenium ca. 100 μm high; ascospores 40–68 × 2.5–3 μm; atranorin present	***B.schweinitzii***
–	Proper exciple without crystals; hymenium less than 70 μm high; ascospores less than 50 μm long; without substance	**3**
3	Hypothecium colourless to pale blue-green; thallus pale grey to pale brown-grey without green colour	***B.friesiana***
–	Hypothecium colourless to brown, dark red-brown; thallus grey-green to green-brown	**4**
4	Proper exciple with green pigment at rim, pale to colourless downwards; hypothecium K– or K+ green-brown; generally on rock or occasionally on bark or moss	***B.egenula* (*Bacidinaegenula*)**
–	Proper exciple colourless at rim, red-brown to black-brown downwards; hypothecium K+ purple; on bark	***B.subincompta* (*Toniniopsissubincompta*)**
5	On rock	**6**
–	On bark or wood	**12**
6	Apothecia pruinose	**7**
–	Apothecia not pruinose	**8**
7	Thallus coarsely granular without forming soredia; apothecia 0.7–1.2 mm diam.; hymenium 70–100 μm high; hypothecium colourless to pale yellow or pale orange; ascospores 40–70 × 2.5–3 μm, 3- to 7-septate	***B.rubella***
–	Thallus granular with soredia; apothecia 0.3–0.7 mm diam.; hymenium 40–50 μm high; hypothecium orange-brown to dark red-brown; ascospores 24–46 × 1–2 μm, 1- to 3-septate	***B.arnoldiana* (*Bacidinaarnoldiana*)**
8	Disc brown, red-brown to black; hypothecium pale brown to dark brown	**9**
–	Disc pale yellow, pale orange to dark brown; hypothecium colourless to pale yellow or pale orange	**10**
9	Proper exciple dark coloured; ascospores 25–35 × 6–10 μm, with L/W ratio less than 10	***B.hakonensis***
–	Proper exciple colourless to pale brown; ascospores 24–46 × 1–2 μm, with L/W ratio over 10	***B.arnoldiana* (*Bacidinaarnoldiana*)**
10	Thallus rimose, wrinkled or warted, but not granular; disc pale yellow or pale grey; epihymenium K–	***B.chloroticula* (*Bacidinachloroticula*)**
–	Thallus granular; disc pale to dark brown; epihymenium K+ purple	**11**
11	Thallus granular forming isidia- or coral-like structures; prothallus absent; apothecia flat; ascospores 25–34 × 1.1–1.9 μm; occasionally on old wood	***B.egenuloidea* (*Bacidinaegenuloidea*)**
–	Thallus granular-warted; white prothallus present on border; apothecia flat to convex; ascospores 24–43 × 2–2.5 μm	***B.inundata* (*Bacidinainundata*)**
12	On wood. Thallus granular forming isidia- or coral-like structures; disc pale orange to dark purple-brown; proper exciple orange-brown to brown at rim; on old wood, but generally on rock	***B.egenuloidea* (*Bacidinaegenuloidea*)**
–	On bark	**13**
13	Proper exciple with radiating clusters of crystals; white pruina present; atranorin present as a major compound or a trace	**14**
–	Proper exciple without crystals; pruina absent; without substance	**17**
14	Hypothecium brown-orange to dark brown; apothecial section K+ purple-red	***B.polychroa***
–	Hypothecium colourless to pale yellow or pale orange; apothecial section K–	**15**
15	Thallus generally coarsely granular, pale grey to green-grey; prothallus white to pale grey when present; ascospores up to 9-septate	***B.rubella***
–	Thallus smooth, wrinkled, warted or granular locally, white-grey to grey; prothallus absent; ascospores up to 13-septate	**16**
16	Thallus grey; disc not pruinose generally, but sometimes white-pruinose; proper exciple with radiating clusters of minute crystals (ca. 0.5 μm wide); epihymenium without distinct colour; ascospores 50–85 × 2.6–3.4 μm	***B.fraxinea***
–	Thallus whitish pale grey; disc light to heavily pruinose; proper exciple with radiating clusters of coarse crystals (up to 10 μm wide); epihymenium brown to dark brown; ascospores 45–70 × 2.5–4.5 μm	***B.suffusa***
17	Thallus granular with soredia-like goniocysts	**18**
–	Thallus smooth, wrinkled, warted or rarely granular, but without soredia	**19**
18	Hypothecium colourless; conidia curved without hook	***B.delicata* (*Bacidinadelicata*)**
–	Hypothecium orange-brown to dark red-brown; conidia hooked	***B.sulphurella* (*Bacidinasulphurella*)**
19	Disc purple-brown to black or slightly blackish when mature; epihymenium K+ purple	**20**
–	Disc pale yellow, pale grey or pale brown; epihymenium K–	**22**
20	Proper exciple colourless to pale yellow at rim; thallus olive-green; apothecia generally pale yellow to pale orange with slightly blackish pigment; epihymenium colourless with a little pale yellow-brown pigment	***B.fuscopallida***
–	Proper exciple dark brown to black-brown at rim; thallus white to pale grey; apothecia purple-brown to black; epihymenium brown to dark brown	**21**
21	Brown pigment of epihymenium deposited in caps of paraphysial tips; thallus wrinkled or warted, but not squamulose; prothallus blackish on border when present; ascospores 32–67 × 2.5–4.5 μm, 3- to 15-septate	***B.heterochroa***
–	Brown pigment of epihymenium distributed in upper hymenial jelly; thallus wrinkled or warted, sometimes squamulose to varnish-like crust; prothallus white between areoles; ascospores 45–80 × 2–3.5 μm, 7- to 28-septate	***B.laurocerasi***
22	Thallus rimose, wrinkled or warted; apothecia ca. 0.2 mm diam.; hypothecium colourless; ascospores 24–28 × 1–1.2 μm, 0- to 3-septate; occasionally on rock	***B.chloroticula* (*Bacidinachloroticula*)**
–	Thallus granular to smooth; apothecia 0.4–1.4 mm diam.; hypothecium straw, yellow-brown to red-brown; ascospores 45–70 × 1.5–4 μm, 3- to 15-septate	**23**
23	Proper exciple yellow-brown to brown at rim; epihymenium yellow-brown; ascospores 1.5–2.5 μm wide, 3- to 7-septate	***B.arceutina***
–	Proper exciple colourless to pale yellow at rim; epihymenium colourless; ascospores 2–4.5 μm wide, 3- to 15-septate	***B.ekmaniana***

## Supplementary Material

XML Treatment for Bacidia fuscopallida

XML Treatment for Bacidia ekmaniana

XML Treatment for Bacidia friesiana

XML Treatment for Bacidia heterochroa

XML Treatment for Bacidia suffusa
